# Allium Vegetables, Garlic Supplements, and Risk of Cancer: A Systematic Review and Meta-Analysis

**DOI:** 10.3389/fnut.2021.746944

**Published:** 2022-03-23

**Authors:** Qifan Zhang, Qing Zhao, Yan Shen, Fuping Zhao, Yan Zhu

**Affiliations:** ^1^Department of Neurology, People's Hospital of Ningxia Hui Autonomous Region, Yinchuan, China; ^2^Department of Pulmonary and Critical Care Medicine, Xi'an No.3 Hospital, Xi'an, China

**Keywords:** cancer risk, supplement, epidemiology, meta-analysis, allium vegetable

## Abstract

**Purpose:**

The role of allium vegetables or garlic supplements on reducing cancer risk was inconsistent between laboratory study findings and related epidemiologic studies.

**Methods:**

Studies assessing the effect of allium vegetables and garlic supplement consumption on cancer risk were included in our meta-analysis. We used fixed- or random-effects models to pool effect measures to evaluate the highest and lowest consumption. A dose-response regression analysis was used to assess the association between allium vegetables, garlic supplements, and cancer risk.

**Results:**

In a pooled analysis of 22 studies with 25 reports on allium vegetables, a high consumption of allium vegetables showed no significant association with cancer risk (relative risk [RR] = 0.97, 95% confidence interval [CI] 0.92–1.03) in a fixed-effects model. Similarly, garlic supplements were not found to be significantly associated with an increased risk of cancer (RR = 0.97, 95% CI 0.84–1.12) in a random-effects model involving a pooled analysis of 10 studies with 11 reports. Consumption of allium vegetables did not significantly correspond with cancer risk (*P* for nonlinearity = 0.958, *P* for linearity = 0.907).

**Conclusion:**

In this meta-analysis, we found no evidence that higher consumption of allium vegetables or garlic supplements reduced the risk of cancer; however, this finding requires further validation.

**Systematic Review Registration:**

https://www.crd.york.ac.uk/prospero/#recordDetails, identifier: CRD42021246947.

## Introduction

The burden of cancer incidence and mortality is rapidly increasing worldwide. According to estimates from the World Health Organization (WHO), there were 19.3 million new cancer cases and almost 10.0 million cancer deaths in 2020 (https://gco.iarc.fr/today/home). Over the past decade, the search for dietary factors with effects on cancer prevention have led to the rapid expansion of the field of dietary patterns and cancer. Studies of individual nutrients and phytochemicals have revealed associations between certain dietary factors and cancer risk (1). Allium vegetables, mainly garlic, onion, and leeks, contained a high level of organosulfur compounds and flavonoids. Evidence from laboratory studies indicates that allium vegetables have anticancer effects through a variety of pathways (2–5). Therefore, allium vegetable consumption has been regarded as a potential dietary strategy for preventing pan-cancer. However, clinical findings examining the effects of allium vegetables on cancer risk reduction have been inconsistent. Previous systemic reviews and meta-analyses have shown that high consumption of allium vegetables might have a protective effect against cancer (6, 7). However, other studies have found no significant association between allium vegetable consumption and risk of cancer (7, 8).

Garlic supplements, as the most widely used type of allium vegetable-related supplements, were evaluated in the majority of previous meta-analyses. However, some studies have reported that garlic supplements were associated significantly with an increased risk of cancer growth (8, 9). Recently, an additional trial (10) with long-term follow-up assessed the effects of garlic supplements on cancer risk but did not detect any beneficial the benefits of garlic supplements on gastric cancer incidence.

Moreover, most previously published meta-analyses have focused on gastrointestinal cancers, like colorectal and stomach cancer (8, 11). However, to the best of our knowledge, no meta-analysis has comprehensively evaluated the role of allium vegetables or garlic supplements in total cancer prevention. Due to this conflicting evidence, as well as the limitations of previous reviews and the availability of new findings, the systematic review and meta-analysis was conducted to evaluate the effects of allium vegetables and garlic supplements on pan-cancer risk.

## Methods

### Protocol and Guidance

This study was performed in accordance with the Preferred Reporting Items for Systematic Reviews and Meta-Analysis (PRISMA) (12). The protocol for this review was registered with PROSPERO (CRD42021246947).

### Inclusion and Exclusion Criteria

After preliminary searches, we decided to include prospective studies, and took the pooled analysis of retrospective studies as our next work. Thus, we only considered trials to be eligible if they involved prospective study designs, including cohort, case-cohort, or intervention studies; if they investigated the relationship between allium vegetables or garlic supplements and malignancy risk; and if they provided or allowed for the calculation of relative risks (RR) with 95% confidence intervals (CIs).

We excluded studies if they were designed as nonhuman studies, case reports, or case series or had a retrospective study design, such as a case-control study; if they were editorials, or letters without the analyses of primary data; or, if they did not provide sufficient data; if they did not use an analytic epidemiologic design.

### Data Sources and Searches

One of the authors (Q.F. Zhang) performed the search of several databases: Medline, Embase, and Web of Science from inception to May 1, 2021. The following combined search terms were used: MeSH headings and keywords relating to “neoplasms,” the MeSH heading “allium,” and keywords related to the allium vegetables and garlic supplements. Ongoing or unpublished eligible studies were also searched through ClinicalTrials.gov and the World Health Organization International Clinical Trials Registry Platform (WHO ICTRP). To maximize the identification of relevant studies, the reference lists of eligible studies were also reviewed. If multiple studies remained with the same or partially overlapping populations, we extracted the data with the largest sample size or the longest follow-up duration. If duplicate studies offered results related to different outcomes, we included them all in the pooled analysis of specific outcomes. An English-language restriction was also included in our search strategy.

Two researchers (Q. F. Zhang and Q. Zhao) independently screened all titles and abstracts of studies meeting the inclusion criteria. They then obtained full-text versions and performed further screening if studies were deemed eligible. We resolved any disagreements by consensus.

### Data Extraction and Quality Assessment

Two independent researchers (Q.F. Zhang and Y. Shen) used a standard form to extract data from the included studies, including first author, publication year, study design, geographic region, sample size (number of cases and total participants), duration of follow-up, demographics of participants, tumor site, exposure, and effect sizes with 95% CIs of highest vs. lowest consumption category. We extracted effect sizes that represented the greatest degree of control for potential confounders. If person-years were not presented, they were using the number of subjects multiplied by the mean duration of follow-up. When a study reported an outcome of interest but without estimates, we tried to contact the author for related data. We resolved any disagreements by consensus.

Two researchers (Y. Shen and F.P. Zhao) independently assessed the quality of included studies by the Newcastle-Ottawa Scale (NOS; http://www.ohri.ca/programs/clinical_epidemiology/oxford.asp). Publication bias was assessed qualitatively by visual estimation of funnel plots and quantitatively by calculation of Egger's test (13). We considered *P*-value less than to represent the possibility of small-study effects.

### Data Synthesis and Analysis

The primary outcome for this meta-analysis was the incidence of cancer. We considered the RRs and 95% CI as the effect sizes for all studies. The association between allium vegetables/garlic supplements and cancer risk was quantified by pooling the RRs for the highest vs. lowest consumption category using random-effects or fixed-effects models according to the extent of between-study heterogeneity. The extent of heterogeneity was quantified by the *Q* test (14) and I^2^ score (15). If significant heterogeneity was not observed (I^2^ < 50%), we used a fixed-effects model to pool outcomes; otherwise, a random-effects model was used (I^2^ ≥ 50%).

### Dose-Response Meta-Analysis

To precisely assess the effect of allium vegetable consumption on cancer risk, a random-effects dose-response meta-analysis was performed, as described previously (16). Moreover, we used the restricted cubic splines with four knots at fixed percentiles (5%, 35%, 65%, and 95%) of the distribution to assess the potential curve-linear relationship between allium vegetable consumption and cancer risk (17). For each study, we used the mean or median level of each category consumption for assigning to each corresponding RR. If data were not available, we assigned the midpoint of the upper and lower boundaries of each category as the average consumption.

### Meta-Regression, Subgroup, and Sensitivity Analyses

Meta-regression and subgroup analyses were performed to identify potential sources of heterogeneity. We stratified included studies into different subgroups according to the number of cases, sex, location, follow-up period, allium vegetable species, and tumor sites. We conducted further sensitivity analyses by excluding one study per iteration. Because the probability of false-negative results is rather high after the adjustment of multiple testing, we did not adjust p values in the present meta-analysis.

## Results

### Eligible Studies and Study Characteristics

The search and selection procedures of literature are presented in Supplementary Figure 1. Finally, twenty-two studies on allium vegetables involving 13,677 patients and 10 studies on garlic supplements involving 6,555 patients were included in this meta-analysis. The main characteristics of included studies are summarized in Table 1.

**Table 1:** Characteristics of studies included in the meta–analysis.

**Author**	**Year**	**Study design**	**Location**	**Cases**	**Total participants**	**Follow–up period (years)**	**Sex**	**Age at baseline**	**Tumor site**	**Exposure**	**Effect Size (95%CI) Highest vs lowest consumption**
Giovannucci (18)	1994	Cohort	USA	205	47,949	6	Male	40–75	Colon	Garlic	0.77 (0.51–1.16)
Dorant (19)	1994	Case-cohort	Netherlands	484	120,852	3.3	Both		Lung	Onion	0.80 (0.52–1.24)
										Leek	1.08 (0.80–1.45)
										Garlic supplements	1.22 (0.81–1.86)
Steinmetz (20)	1994	Cohort	USA	212	41,837	5	Female	55–69	Colon	Garlic	0.68 (0.46–1.02)
Dorant (21) (1)	1996	Case-cohort	Netherlands	152	120,852	3.3	Both	55–69	Stomach	Garlic supplements	1.29 (0.62–2.67)
				139					Stomach	Onion	0.50 (0.26–0.95)
										Leek	0.69 (0.42–1.14)
Dorant (22) (2)	1996	Case-cohort	Netherlands	443	120,852	3.3	Both	55–69	Colon	Garlic supplements	1.36 (0.79–2.35)
				(293 colon, 150 rectum)				Rectum		1.28 (0.63–2.60)
				(243 male, 200 female)			Male		Colon	Onion	0.87 (0.48–1.65)
									Rectum		0.66 (0.28–1.52)
							Female		Colon		1.49 (0.79–2.81)
									Rectum		1.34 (0.55–3.31)
							Male		Colon	Leek	1.10 (0.71–1.70)
									Rectum		0.72 (0.40–1.30)
							Female		Colon		1.18 (0.73–1.89)
									Rectum		1.31 (0.60–2.85)
Schuurman (23)	1998	Case-cohort	Netherlands	610	58,279	6.3	Male	55–69	Prostate	Allium vegetables	0.95 (0.69–1.33)
										Onion	0.93 (0.79–1.10)
										Leek	1.38 (1.08–1.76)
Li (24)	2004	Intervention	China	142	5,033	5	Both	35–74	All	Garlic supplements	0.67 (0.43–1.03)
				(107 male, 35 female)	(2,526 intervention, 2,507 control)				Stomach		0.48 (0.21–1.06)
				(53 stomach, 48 liver)				-	Liver		0.78 (0.35–1.74)
				(63 intervention, 79 control)		Male		All	Garlic supplements	0.51 (0.30–0.85)
									Stomach		0.36 (0.14–0.92)
									Liver		0.71 (0.28–1.78)
							Female		All	Garlic supplements	1.50 (0.62–3.61)
									Stomach		1.14 (0.22–5.76)
									Liver		1.17 (0.23–5.92)
Schulz (25)	2005	Cohort	Europe	581	325,640	6.3 (mean)	Female	19–98	Ovary	Garlic/onion	0.79 (0.62–1.01)
Lin et al. (26)	2006	Cohort	USA	878	107,401	10	Both	30–75	Colorectum	Onion	1.03 (0.82–1.29)
				380	35,425		Male	40–75			0.92 (0.65–1.29)
				498	71,976		Female	30–55			1.13 (0.84–1.53)
Boeing (27)	2006	Cohort	Europe	352	345,904	5.8	Both	NA	Upper aero-digestive tract	Garlic/onion	1.17 (0.73–1.86)
				255	130,633		Male				1.04 (0.79–1.36)
				97	215,271		Female				1.20 (0.73–1.97)
Gonzalez (28)	2006	Cohort	Europe	65	481,518	6.5	Both	35–70	Esophagus	Garlic/onion	1.27 (0.59–2.73)
Larsson (29)	2006	Cohort	Sweden	139	82,002	7.2 (mean)	Both		Stomach	Allium vegetables	0.90 (0.58–1.41)
					(36,664 female, 45,338 male)						
Kirsh (30)	2007	Cohort	USA	1,338	29,361	4.2 (mean)	Male	55–74	Prostate	Garlic	0.88 (0.76–1.03)
										Onion	1.01 (0.88–1.17)
Satia (31)	2009	Cohort	USA	428	77,512	5	Both	50–76	Colorectum	Garlic supplements	1.35 (1.01–1.81)
				665	77,125				Lung	Garlic supplements	1.05 (0.83–1.34)
Epplein (32)	2010	Cohort	China	206	73,064	12	Female	40–70	Stomach	Allium vegetables	1.10 (0.74–1.63)
				132	59,247	6	Male	40–74			0.92 (0.58–1.46)
Walter (33)	2010	Cohort	USA	588	66,227	6.5 (mean)	Both	50–76	Hematologic malignancies	Garlic supplements	0.55 (0.34–0.87)
Gonzalez (34)	2011	Cohort	Europe	1,070	299,651	9 (mean)	Female	35–70	Cervix	Garlic and onions	1.09 (0.85–1.40)
Brasky (35)	2011	Cohort	USA	1,602	35,239	6.1 (median)	Men	50–76	Prostate	Garlic supplements	1.00 (0.85–1.17)
Steevens (36)	2011	Case-cohort	Netherlands	824	120,852	16.3	Both	55–69	Esophageal squamous cell carcinoma	Allium vegetables	1.64 (0.88–3.08)
									Esophageal adenocarcinoma		0.84 (0.50–1.43)
									Gastric cardia adenocarcinoma		1.55 (0.94–2.56)
									Gastric noncardia adenocarcinoma		0.97 (0.69–1.35)
									Esophageal squamous cell carcinoma	Onion	1.10 (0.79–1.53)
									Eesophageal adenocarcinoma		0.84 (0.65–1.09)
									Gastric cardia adenocarcinoma		1.21 (0.95–1.53)
									Gastric noncardia adenocarcinoma		0.91 (0.78–1.06)
McCullough (9)	2012	Cohort	USA	1,130	99,700	7	Both	69	Colorectum	Garlic	1.03 (0.77–1.37)
				579	42,824		Male				1.19 (0.79–1.79)
				551	56,876		Female				0.87 (0.58–1.32)
				764	89,921		Both		Colorectum	Garlic supplements	1.03 (0.74–1.44)
				390	38,053		Male				0.94 (0.57–1.53)
				374	51,868		Female				1.09 (0.69–1.72)
Gonzalez (37)	2012	Cohort	Europe	683	477,312	11.02 (mean)	Both	35–70	Stomach	Garlic/onion	0.97 (0.76–1.25)
Meng (38)	2013	Cohort	USA	1,339	76,208	24	Female	30–55	Colorectum	Garlic	1.21 (0.94–1.57)
				1,029	45,592	22	Male	40–75			1.00 (0.71–1.42)
				578	76,208	4	Female	30–55	Colorectum	Garlic supplements	0.72 (0.49–1.07)
				559	45,592	10	Male	40–75			1.22 (0.83–1.78)
Vogtmann (39)	2013	Cohort	China	398	61,274	6.3 (median)	Men	40–74	Colorectum	Allium	0.99 (0.77–1.27)
Maasland (40)	2015	Case-cohort	Netherlands	415	120,852	20.3	Both	55–69	Head and neck	Allium	1.07 (0.76–1.50)
										Onion	0.93 (0.77–1.11)
Kim (41)	2018	Cohort	USA	292	123,484		Both		Stomach	Garlic	1.39 (0.89–2.17)
				138	77,086	30	Female				1.34 (0.72–2.47)
				154	46,398	28	Male				1.45 (0.76–2.78)
Li (10)	2019	Intervention	China	150	3365	14.7	Both	35–64	Stomach	Garlic supplements	0.81 (0.57–1.13)
					(1678 intervention, 1687 control)						
Yu (42)	2020	Cohort	China	270	3199	10	Both	30–70	Liver	Garlic	0.62 (0.42–0.93)

The assessment of risk of bias and quality showed that all included studies had NOS scores of ≥7, indicting a lower risk of bias and better quality.

### Highest vs. Lowest Consumption Category

**Figure 1:**
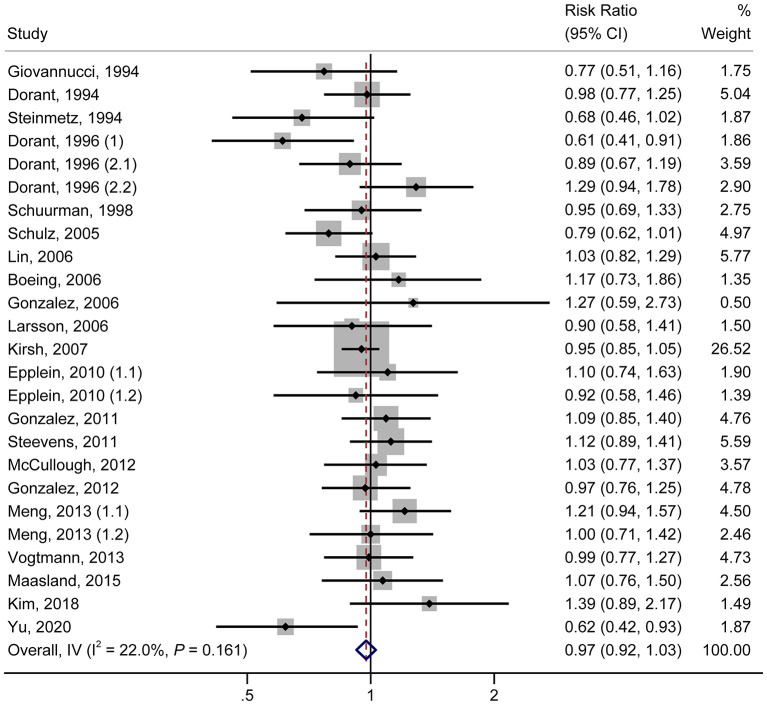
Forest plot of the association between allium vegetable consumption and cancer risk (highest vs. lowest category of allium vegetable consumption).

**Figure 2:**
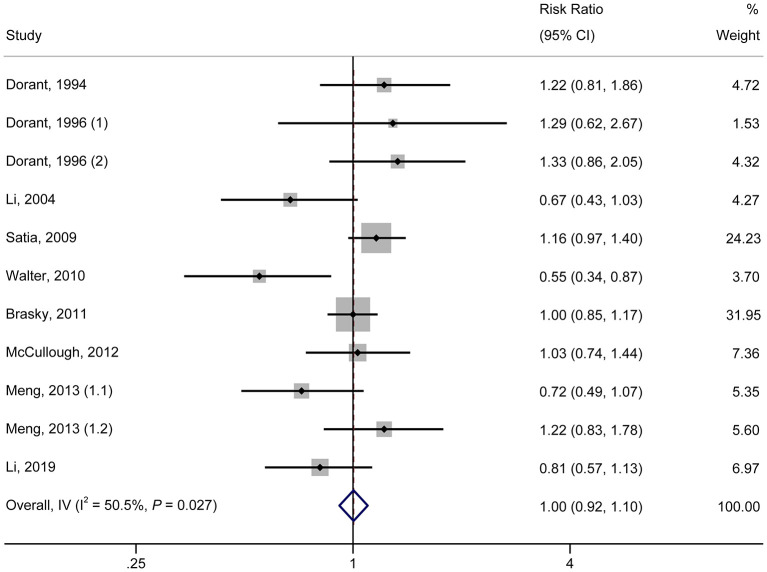
Forest plot of the association between garlic supplement consumption and cancer risk (highest vs. lowest category of supplement consumption).

As shown in Figure 1, in the pooled analysis of the 22 studies (9, 18–23, 25–30, 32, 34, 36–42) with 25 reports on allium vegetable consumption, no significant association was observed between a high consumption of allium vegetables and cancer risk (RR, 0.97; 95% CI, 0.92–1.03; *P* = 0.356; I^2^ = 22.0%; *P*_h_ = 0.161). Garlic supplements were also not found to be significantly associated with an increased risk of cancer (RR, 0.97; 95% CI, 0.84–1.12; *P* = 0.705; I^2^ =50.5%; *P*_h_ = 0.027) in a random-effects model involving a pooled analysis of 10 studies (9, 10, 19, 21, 22, 24, 31, 33, 35, 38) with 11 reports (Figure 2).

### Dose-Response Meta-Analysis

**Figure 3:**
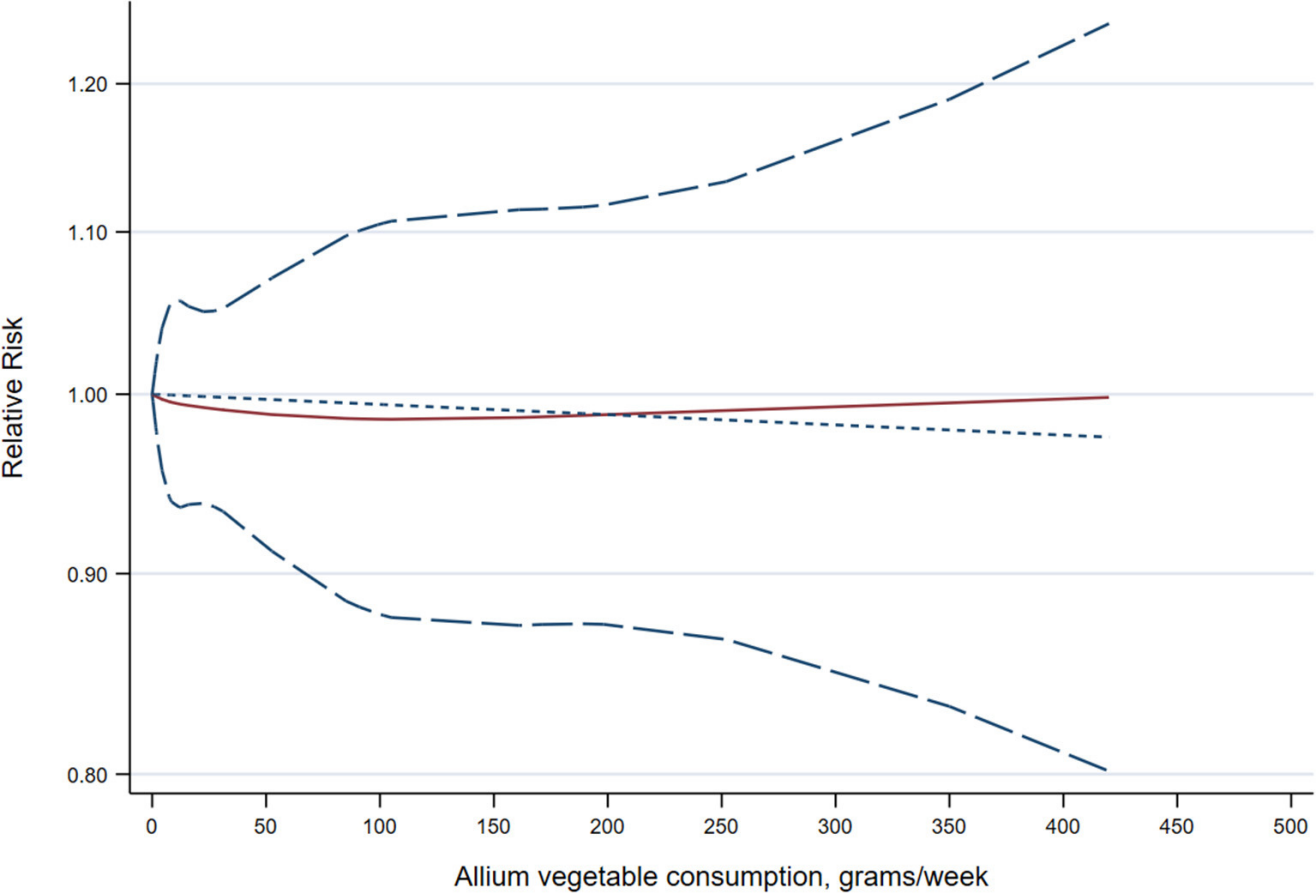
Dose-response meta-analysis of the association between allium vegetable consumption and cancer risk. The solid line represents the linear relationship, and the short-dashed line represents the nonlinear relationship, with the long-dashed lines representing the 95% confidence intervals for the spline model.

A random-effects, dose-response meta-analysis was performed to precisely assess the relationship between allium vegetable consumption and risk of cancer. A total of 14 studies (9, 19, 21–23, 26, 29, 30, 32, 38–42) with 20 reports were included. We did not find a curvilinear association between allium vegetable consumption and risk of cancer (*P* = 0.958 for nonlinearity; *P* = 0.907 for linearity) (Figure 3).

### Subgroup Analysis

In an analysis of individual allium vegetables, garlic intake was investigated in seven studies (9, 18, 20, 30, 38, 41, 42) with eight reports, which showed no association with cancer risk (RR, 0.93; 95% CI, 0.79–1.09). An analysis of eight studies (19, 21–23, 26, 30, 36, 40) with nine reports related to onion consumption showed that a high amount of onion consumption was not associated with cancer risk (RR, 0.96; 95% CI, 0.90–1.03). Moreover, four studies (19, 21–23) with five reports investigated the consumption of leeks and risk of cancer, with the pooled analysis showing no association between leek consumption and cancer risk (RR, 1.08; 95% CI, 0.88–1.34) (Table 2). Another subgroup analysis based on tumor site showed that allium vegetable consumption was not significantly associated with the risk of cancer in the digestive tract (RR, 1.01; 95% CI, 0.94–1.09) or the non-digestive tract (RR, 0.94; 95% CI, 0.87–1.01).

**Table 2:** Subgroup analysis of the association between allium vegetable and supplement consumption and cancer risk.

**Subgroup**	**No of reports**	**RR (95% CI)**	***P* for test**	**I^**2**^ (%)**	***P* for heterogeneity**	***P* for meta–regression**
***Allium vegetables***						
Overall	25	0.97 (0.92–1.03)	0.356	22.0	0.161	
No of cases						
≥500	10	0.99 (0.93–1.06)	0.775	0.0	0.516	0.433
<500	15	0.95 (0.86–1.04)	0.238	36.3	0.079	
Sex						
Female	10	1.03 (0.93–1.14)	0.539	38.0	0.105	
Male	11	0.96 (0.89–1.04)	0.313	0.0	0.918	0.290
Location						
Europe and America	21	0.98 (0.93–1.04)	0.523	20.9	0.190	0.490
China	4	0.92 (0.77–1.09)	0.314	38.8	0.179	
Follow-up period						
≥7 y	12	1.05 (0.96–1.14)	0.304	0.0	0.455	0.063
<7 y	13	0.93 (0.87–1.00)	0.040	22.5	0.216	
Type of intake						
Garlic	8	0.93 (0.79–1.09)	0.363	54.9	0.030	
Onion	9	0.96 (0.90–1.03)	0.285	7.8	0.370	0.637
Leek	5	1.08 (0.88–1.34)	0.445	46.5	0.112	0.114
Tumor site						
Digestive tract	18	1.01 (0.94–1.09)	0.791	18.1	0.237	
Non-digestive tract	7	0.94 (0.87–1.01)	0.110	27.1	0.222	0.251
***Garlic supplements***						
Overall	11	0.97 (0.84–1.12)	0.705	50.5	0.027	
No of cases						
≥500	5	0.96 (0.80–1.16)	0.689	59.9	0.029	0.840
<500	6	0.99 (0.75–1.31)	0.957	47.4	0.107	
Sex						
Female	3	0.95 (0.65–1.39)	0.783	37.8	0.200	
Male	4	0.92 (0.69–1.22)	0.569	59.4	0.060	0.885
Location						
Europe and America	9	1.03 (0.88–1.19)	0.727	46.2	0.062	0.141
China	2	0.75 (0.58–0.99)	0.040	0.0	0.503	
Follow-up period						
≥5 y	7	0.94 (0.79–1.12)	0.473	58.6	0.025	0.475
<5 y	4	1.07 (0.78–1.47)	0.661	45.7	0.137	
Tumor site^*^						
Digestive tract	8	1.02 (0.83–1.26)	0.859	51.0	0.046	
Non-digestive tract	5	0.95 (0.78–1.17)	0.639	47.1	0.109	0.638

* *Two studies reported effect sizes of more than one disease subgroup*.

The association between allium vegetable consumption and cancer risk were similarly nonsignificant in other subgroup analyses based on the number of cases, sex, and location. However, a stratified analysis based on the duration of follow-up period indicated that a high amount of allium vegetable consumption was marginally associated with a decreased risk of cancer in patients with a follow-up period of less than 7 years (RR, 0.93; 95% CI, 0.87–1.00) (Table 2).

Moreover, in the subgroup analysis of garlic supplements, the consumption of garlic supplements was not associated with cancer risk according to the number of cases, sex, follow-up period, or tumor site. Stratified by study location, however, garlic supplement consumption was marginally associated with a decreased cancer risk in Chinese patients (RR, 0.75; 95% CI, 0.58–0.99); however, only two reports were pooled in this subgroup.

### Sensitivity Analysis

**Figure 4:**
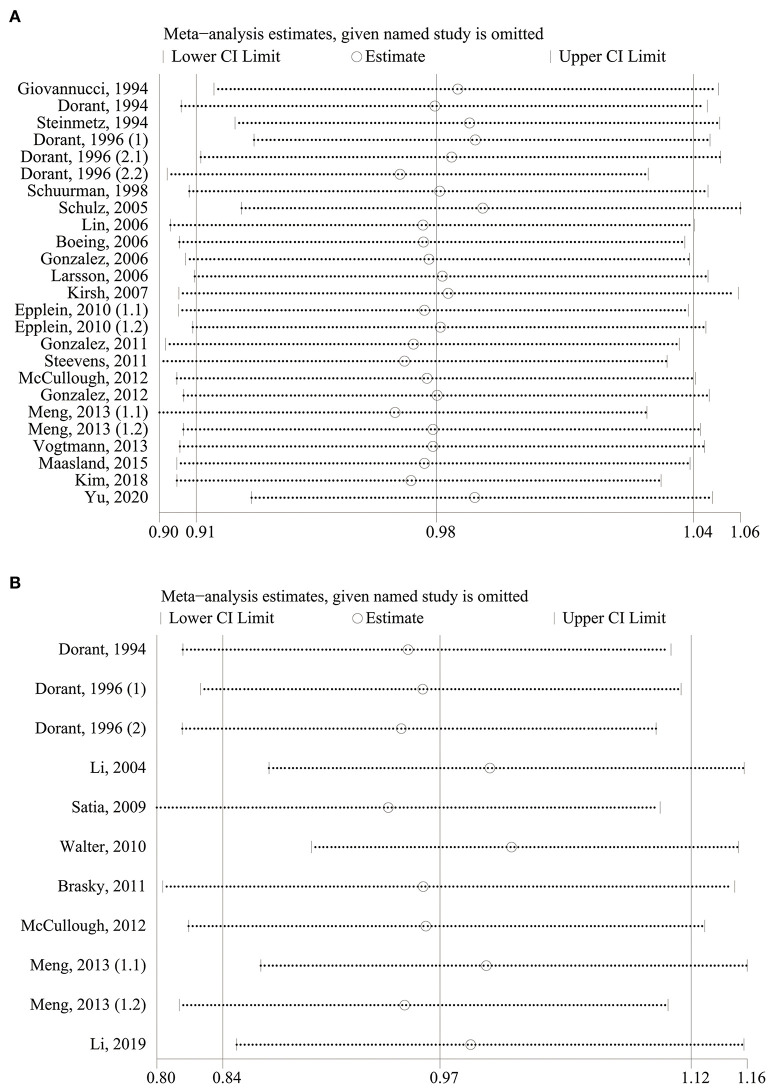
Sensitivity analysis for cancer risk with allium vegetable **(A)** and garlic supplement **(B)**.

As shown in Figure 4, in our sensitivity analysis, the specific RRs from 25 reports for allium vegetable consumption ranged from a low of 0.96 (95% CI, 0.91–1.02; I^2^ = 17.6%; *P*_h_ = 0.219) after omitting the report by Meng, 2013 (1.1) (38) to a high of 0.99 (95% CI, 0.93–1.04; I^2^ = 17.2%; *P*_h_ = 0.225) after omitting the report by Schulz, 2005 (25), but they were, in general, similar. The 11 report-specific RRs for garlic supplement intake ranged from a low of 0.96 (95% CI, 0.86–1.06; I^2^ = 47.2%; *P*_h_ = 0.048) after omitting the report by Satia, 2009 (31) to a high of 1.03 (95% CI, 0.94-1.13; I^2^ = 34.2%; *P*_h_ = 0.134) after omitting the report by Walter, 2010 (33), which were again similar. The results of sensitivity analysis showed that there was no significant variation in the pooled RR after excluding any one of the studies, proving the stability of the overall results.

### Meta-Regressions

Our univariate meta-regression analysis found no statistically significant differences, revealing that the analyzed stratification factors were not a source of heterogeneity. However, we did find that the cancer risk was significantly higher in studies with allium vegetable consumption and a longer duration of follow-up (*P* for interaction = 0.017; Supplementary Figures 2, 3).

### Publication Bias

No publication bias was observed in this meta-analysis. No asymmetry were found through a funnel plot analysis. Moreover, Egger's test (*P* = 0.910 for allium vegetables; *P* = 0.426 for garlic supplements) detected no significant small-study effects (Supplementary Figures 4, 5).

## Discussion

### Principal Findings

To our knowledge, the current meta-analysis was the first to assess the effects of allium vegetable and supplement consumption on pan-cancer risk. In our findings, there was no significant effect of higher consumption of allium vegetables on cancer risk. Furthermore, results from a dose-response meta-analysis revealed no nonlinear or linear relationship between allium vegetable consumption and cancer risk. Meanwhile, a subgroup analysis in patients with a follow-up duration of less than 7 years showed that a higher amount of allium vegetable consumption was marginally related to a decreased cancer risk. Our meta-regression analysis also revealed that the follow-up period might be a potential source of heterogeneity. Neither garlic and onion, nor leek consumption were associated with cancer risk. We also found that garlic supplements were not significantly associated with cancer risk. Our findings do not support the conclusion that allium vegetable or supplement intake are protective factors against cancer. Our comprehensive cancer risk assessment based on the consumption of allium vegetables or supplements enables a general understanding of the association between allium vegetable and supplement consumption and cancer risk.

### Comparison With Other Studies

Many previous meta-analyses have focused on the association between allium vegetable consumption and risk of gastrointestinal cancers, including colorectal cancer (8, 43) and stomach cancer (6, 44) with inconsistent findings. In 2014, two meta-analyses involving observational studies found a higher consumption of allium vegetables was not associated with colorectal cancer risk (8, 43). Similar nonsignificant results was found in a meta-analysis assessing the effects of allium vegetables on esophageal cancer (7). However, consumption of a large amount of allium vegetables was found to reduce the risk of stomach cancer based on data mainly derived from case-control studies (6, 44). In our pooled analysis of only prospective studies, we found that higher allium vegetable consumption did not reduce the risk of cancer. A subgroup analysis also showed consistently nonsignificant results for different tumor sites, including the digestive tract and non-digestive tract. Though our study did suggest that higher allium vegetable consumption may have a protective role against cancer risk in patients with a follow-up duration of less than 7 years, the statistical difference was marginal for this finding. Our meta-regression analysis also found that cancer risk was significantly higher in studies with allium vegetable consumption in patients with a longer duration of follow-up. The follow-up period may therefore explain the heterogeneity in the pooled RR. We hypothesize that a longer follow-up period may have led to the development of cancer in more patients. Overall, allium vegetable consumption was not associated with cancer risk in the current meta-analysis.

Findings related to garlic, the mostly widely analyzed individual allium vegetable item, and its role in cancer risk has varied across studies and cancer types. A meta-analysis of 18 studies in 2018 revealed that high garlic consumption was associated with a reduced gastric cancer risk (11). Furthermore, its protective effect has been proven for upper aerodigestive tract cancer (7), esophageal cancer (7), and prostate cancer (45). However, conclusions from other meta-analyses did not support a protective role of garlic consumption in head and neck cancer (7), colorectal cancer (46). It is worth noting that previous meta-analyses always combined garlic and garlic supplements, however, garlic supplements have been proven to be significantly associated with an increased risk of certain cancer types, including colorectal cancer (8). In the current meta-analysis, we conducted a subgroup analysis exploring the role of garlic consumption on cancer prevention without garlic supplements, finding no association between higher garlic consumption and reduced cancer risk after pooling the results of eight reports, including five on colorectal cancer (9, 18–20, 38) (1,2), one on stomach cancer [Kim, 2018 (41)], one on prostate cancer [Kirsh, 2007 (30)], and one on liver cancer[Yu, 2020 (42)]. It is worth exploring whether the addition of retrospective studies, such as case-control studies, would have led to different results. However, these pooled results did not support a protective role of garlic in cancer prevention.

In addition to garlic, other individual allium vegetable items have also been investigated for their effects on cancer risk. For example, onions have been demonstrated to be a protective factor against gastric cancer (6), esophageal cancer (7), and laryngeal cancer (7), but not colorectal (43) or prostate (45) cancers. As with garlic, the current meta-analysis showed no protective effects against cancer for onions or leeks.

Garlic supplements contains various mixture that must be prepared at a proper temperature, which have been demonstrated to be associated with toxicity (47). Accumulating evidence indicates important diversity between garlic supplements and raw/cooked garlic (48). Allicin, as the major biologically active element of garlic, is not present in garlic supplement (49). A previous meta-analysis that included five studies with 11 reports on the effects of garlic supplements showed that use of these supplements was significantly associated with an increased risk of colorectal cancer (8); however, another analysis comprising 10 reports demonstrated no significant association (46). In 2019, a blinded randomized placebo-controlled trial enrolled 3,365 residents in a region at high risk for gastric cancer in China (10). In a report of its long-term results, this study revealed that garlic supplements yielded a statistically significant decrease in gastric cancer mortality but a statistically insignificant decrease in incidence. By including the results of those recently published studies, the current meta-analysis provides an independent estimate of the effects of garlic supplements on cancer risk, showing that these supplements play neither a protective nor a harmful role in cancer. Notably, however, our pooled analysis of two reports from China showed inconsistent results, revealing that garlic supplement may decrease the risk of cancer incidence by 25%. However, we speculate that the small sample size with insufficient power generated less stable results.

Although many systematic reviews have studied the association between allium vegetable consumption and cancer risk, some issues still exist and need to be resolved, especially related to the discrepancies in their findings. First, retrospectively designed studies, such as case-control studies, have been enrolled in some previous meta-analyses. In general, case-control studies are more prone to recall and selection biases and are not entirely controlled for confounders, including those known to be protective factors, which may bias the results (8). Second, dietary factors are generally considered to be mainly related to gastrointestinal cancers, although laboratory evidence indicates that some ingredients in allium vegetables may also exert anticancer effects on other cancer types besides colorectal or stomach cancer (2, 50–52). More epidemiologic studies, especially prospective studies, are required to further verify the role of allium vegetables in non-digestive tract cancers. Third, supplements provide the possibility of conducting randomized controlled trials for assessing the effects of allium vegetable on cancer. However, it remains unclear whether specific ingredients in allium vegetables play the major anticancer role or if supplements play the same role as raw allium vegetables. Both of these factors make the results of previously pooled analyses unreliable. To avoid the influence of these factors, the current meta-analysis included prospectively designed studies and separately pooled allium vegetables and supplements. We believe our findings offer more stable and comprehensive evidence about the role of allium vegetables.

### Strengths and Limitations

This present meta-analysis had several strengths. Because only prospective studies were included, our findings are unlikely to be influenced by recall or selection bias. Also, unlike most previous studies that assessed only specific cancer types, the associations between allium vegetables and multiple cancer types were examined in our study. Additionally, garlic supplements were analyzed separately from allium vegetables due to differences in their properties and effects. Moreover, our results based on pooled estimates were consistent in stratified analyses across various scenarios, indicating the robustness of our findings. Furthermore, we quantified the association between consumption of allium vegetables and cancer risks by performing linear and non-linear dose-response analyses. Finally, our subgroup, publication bias, and sensitivity analyses demonstrated the reliability and stability of the results.

However, some limitations existed the current meta-analysis. First, the measurements of dose level of allium vegetables or garlic supplements (daily, weekly, monthly, or gram, serving, clove) varied across the included studies, making it impossible to accurately compare equivalent consumption. Also, the included studies only reported use or nonuse, leading to the impossibility of assessing the dose-response relationship between garlic supplements and cancer risk. Second, although we pooled effect sizes adjusting for most established risk factors, the causes of cancer incidence are complicated and sometimes unclear, which may have introduced confounders that could have influenced our findings. Third, many laboratory studies have demonstrated the role of garlic components in inhibiting tumor growth and progression, suggesting that they might also play a role in prolonging the survival of cancer patients. However, whether allium vegetables or supplements could reduce cancer mobility was not analyzed in the current study, limiting the implications of our findings. Last, though prospective studies are less likely to be influenced by recall or selection bias, well-conducted retrospective studies provide less-biased evidence. The addition of retrospective studies in the pooled analysis was believed to provide more detailed information and strength the reliability of the findings, which was also our next work.

### Implications

Cancer incidence and mortality is a major burden in most countries worldwide. Currently, not only treatment but also prevention of cancer has been of great concern to prolong lifespan of population. Considering the anticancer effects of certain active compounds present in allium vegetables, such as organosulfur, researchers have shown an increasingly interest in the ability of these substances to prevent cancer. Our findings suggest that neither allium vegetables or supplements have clinically relevant effects on cancer prevention; therefore, there is little evidence to support the idea that allium vegetables or supplements can reduce cancer in incidence. Additional large, randomized controlled trials are needed to confirm these results.

### Conclusions

Overall, the current meta-analysis found that higher consumption of allium vegetables or garlic supplements were not associated with a decrease in cancer risk. Randomized controlled trials or well-designed cohort studies are warranted to confirm these findings.

## Data Availability Statement

The original contributions presented in the study are included in the article/Supplementary Material, further inquiries can be directed to the corresponding author.

## Author Contributions

Material preparation, data collection, and analysis were performed by QifZ, QinZ, YS, FZ, and YZ. The first draft of the manuscript was written by QifZ and QinZ. All authors commented on previous versions of the manuscript, read and approved the final manuscript, and contributed to the study conception and design.

## Conflict of Interest

The authors declare that the research was conducted in the absence of any commercial or financial relationships that could be construed as a potential conflict of interest.

## Publisher's Note

All claims expressed in this article are solely those of the authors and do not necessarily represent those of their affiliated organizations, or those of the publisher, the editors and the reviewers. Any product that may be evaluated in this article, or claim that may be made by its manufacturer, is not guaranteed or endorsed by the publisher.
