# Supplementary material for: Allium Vegetables, Garlic Supplements, and Risk of Cancer: A Systematic Review and Meta-Analysis
Source: Front Nutr. 2022 Mar 23;8:746944. doi: 10.3389/fnut.2021.746944 (PMC8985597; doi:10.3389/fnut.2021.746944)
Supplement: Supplementary file 1 [file Data_Sheet_1.docx]

***Supplementary Material***

**Identification**

**Screening**

**Included**

Records identified from:

Databases (n = 5786)

Registers (n = 13)

Records removed before screening:

Duplicate records removed

(n = 4580)

Records screened

(n = 1119)

Records excluded

(n = 1041)

Reports sought for retrieval

(n = 78)

Reports not retrieved

(n = 41)

Reports assessed for eligibility

(n = 37)

Reports excluded:

Adenomatous polyps (n = 3)

Without risk estimate (n = 2)

Duplicate studies (n = 4)

As a part of vegetables (n = 1)

Studies included in review

(n = 27):

Allium vegetables (n = 22)

Garlic supplements (n=10)

**Supplementary Fig. 1** Flowchart of the literature search and selection procedures


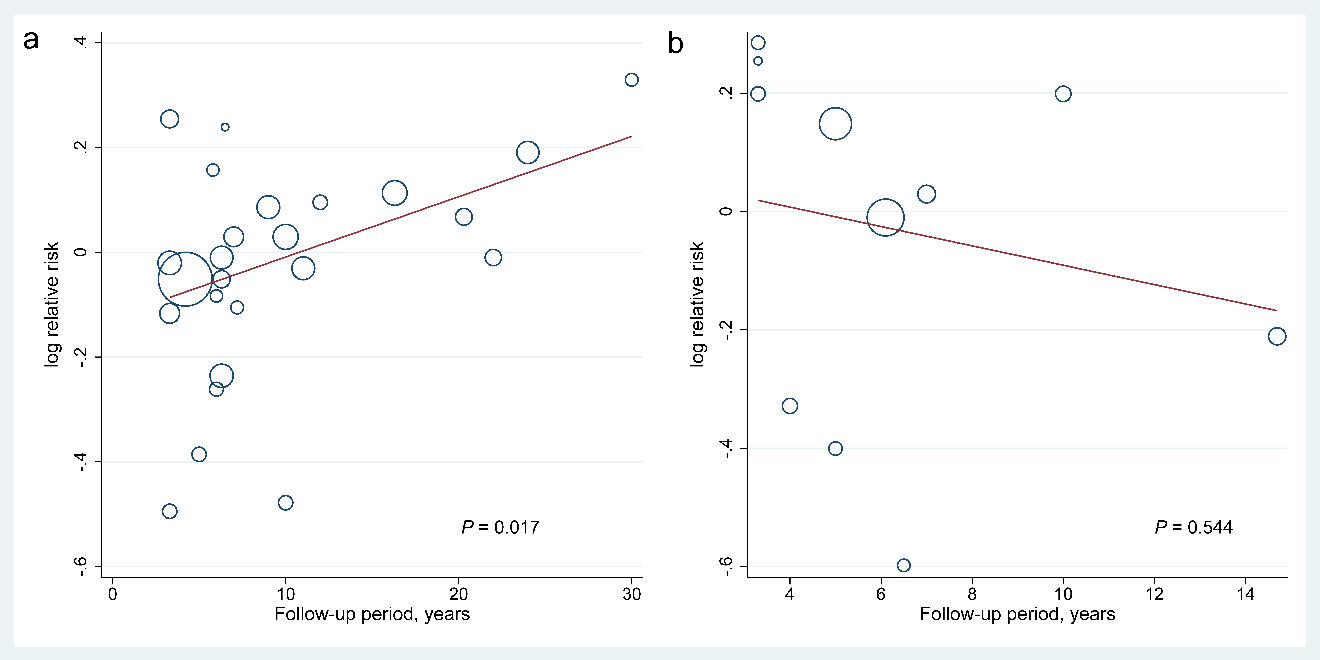


**Supplementary Fig. 2** Meta-regression analysis of cancer risk by length of follow-up with allium vegetable (a) or supplement (b) consumption


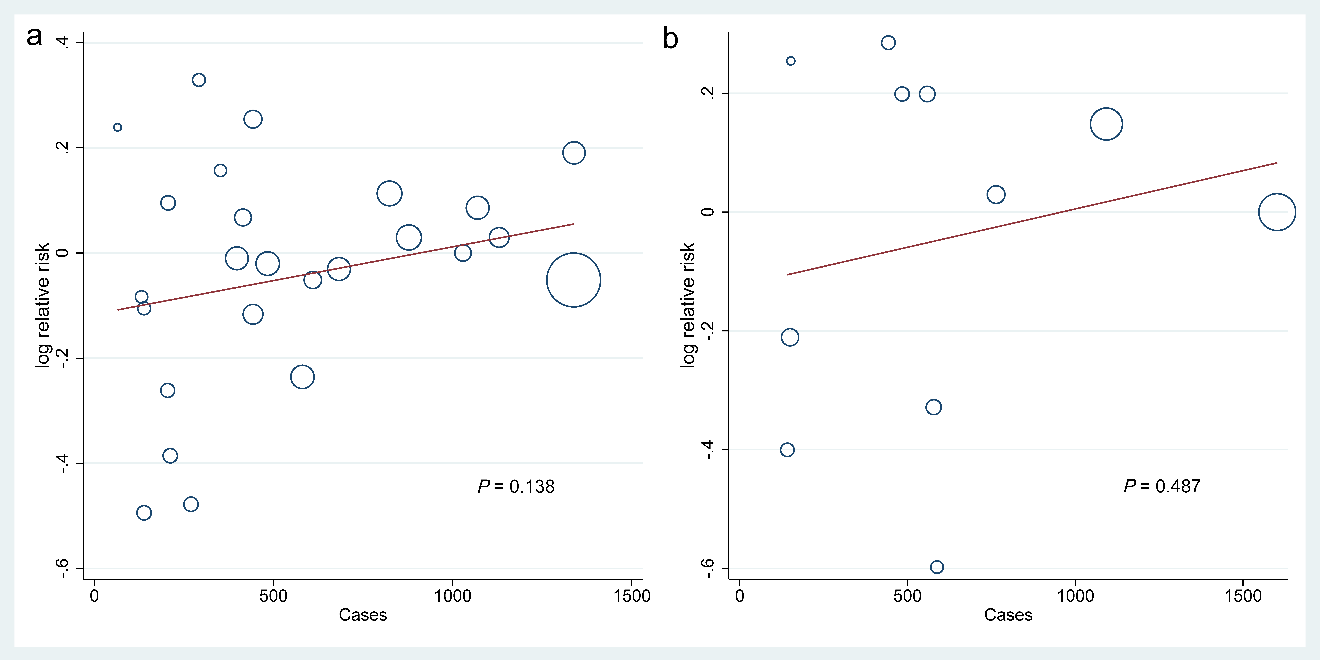


**Supplementary Fig. 3** Meta-regression analysis of cancer risk by size of cases with allium vegetable (a) or supplement (b) consumption


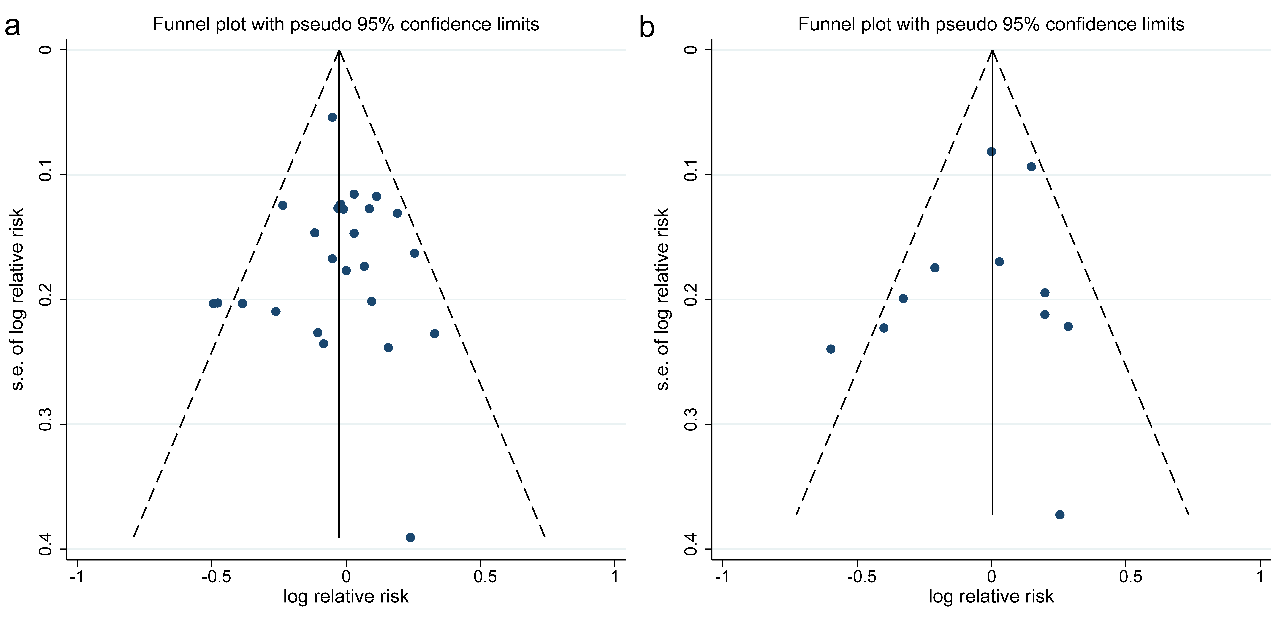


**Supplementary Fig. 4** Assessment of publication bias for cancer risk with allium vegetable (a) or supplement (b) consumption by funnel plot


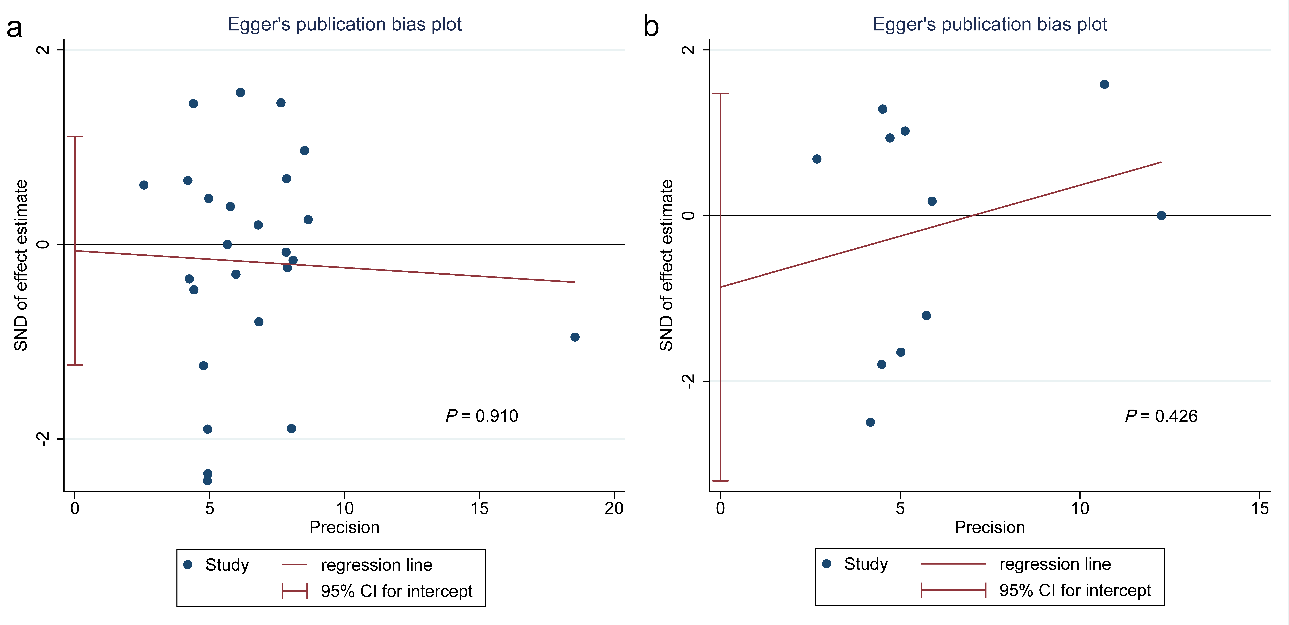


**Supplementary Fig. 5** Assessment of publication bias for cancer risk with allium vegetable (a) or supplement (b) consumption by Egger’s test
